# *Bacillus Cereus* Enhanced Phytoremediation Ability of Rice Seedlings under Cadmium Toxicity

**DOI:** 10.1155/2019/8134651

**Published:** 2019-07-24

**Authors:** Mehmood Jan, Gulmeena Shah, Sadaf Masood, Kamran Iqbal Shinwari, Rashida Hameed, E. S. Rha, Muhammad Jamil

**Affiliations:** ^1^Institute of Crop Science, College of Agriculture and Biotechnology, Zhejiang University, Hangzhou, China; ^2^Department of Biotechnology & Genetic Engineering, Kohat University of Science & Technology, Kohat 26000, Pakistan; ^3^Department of Environmental Science, Zhejiang University, Hangzhou, China; ^4^College of Agriculture and Life Sciences, Sunchon National University, Suncheon 57922, Republic of Korea

## Abstract

Cadmium (Cd^+2^) is a highly toxic metal, which significantly alters different biochemical and metabolic processes in plants. Massive amounts of Cd^+2^ is being released into the environment by different anthropogenic activities. In the present study, plant growth promoting activities of bacterial strain* Bacillus cereus *was evaluated under Cd^+2^ stress in two rice cultivars Basmati-385 and Shaheen Basmati. Cd^+2^ stress significantly decreased plant growth and biomass production in both cultivars. However, with the inoculation of* B. cereus *under Cd^+2^ treatments, reduced Cd^+2^ uptake and increased antioxidant enzymes activities in rice cultivars lead to enhanced plant growth, biomass production, photosynthetic pigments, micronutrients, and lowered electrolytes leakage. This study suggests that* B. cereus* has the ability to alleviating Cd toxicity and increased phytoremediation efficiency of rice seedling under Cd stress.

## 1. Introduction

Cadmium being a toxic pollutant has become a widespread problem in agricultural soils [1–3]. Atmospheric deposition is considered as the main source of cadmium accumulation in the soil; besides this, several anthropogenic activities like industrial waste, irrigation by wastewater, sludge usage and disposition of solid waste are the common sources of Cd introduction into the environment [4]. Cadmium alters many processes in plants like photosynthesis, respiration, plant–water relationship, and mineral nutrition and also interference with the electron-transport chain in growing plants ultimately leading to cause a reduction in crop productivity [5–7]. In addition, Cd^+2^ is classified as a human carcinogen, which may cause cardiovascular disease, skeletal damage, and cancer in the lungs, prostate, and kidneys [8–10]. Therefore, research is needed to avoid Cd^+2^ bioaccumulations in plants and which subsequent entry into the food chain. It is particularly important to reduce Cd^+2^ accumulation in rice grain because rice is a vital cereal crop being utilized as a necessary food source for more than half of the world's population particularly in Asian countries [11–13]. Rice accumulates a higher amount of Cd^+2^ in the grain as compared with other cereals like barley and wheat [14]. To reduce Cd-induced toxicity and uptake, different approaches have been used including priming of seeds with phytohormones/chemicals like salicylic acid, glutathione, brassinosteroids (BRs) phytochelatins (PCs), polyamines (PAs), nitric oxide, abscisic acid, and jasmonic acid [15–18].

However, biopriming techniques have been widely to prevent the toxic effect of pollutant like heavy metals on plant growth and development. Many studies reported that biopriming of seeds with plant growth promoting bacteria (PGPB) or application of PGPB on seedling could enhance plant growth by lowering the uptake of toxic metals in the medium [13, 19, 20]. PGPBs contain 1-aminocyclopropane-l-carboxylic acid (ACC) deaminase efficiently accelerate plant growth by decreasing plant ethylene levels under different stress conditions, such as heavy metals, drought, salinity, and flooding [21]. Bacterial strains also produce bacterial exopolysaccharides (EPSs) which could bind cations and decrease the contents of cations (like Cd) available for the plant uptake. In this way, increasing the population density of EPS-producing bacteria in the root zone could reduce the uptake of cations content, which leads to enhanced plant growth under stress conditions [22]. A wide range of bacterial species which include* Pseudomonas, Bacillus, Arthrobacter, Azotobacter, Enterobacter, Azospirillum, Serratia klebsiella, *and* Alcaligin* could be used for this purpose [23].

Many* Bacillus* species have been used for phytoremediation and also as a protectant from toxic effects of heavy metals. For enhancement of plant growth, these species are directly involved in increased uptake of nitrogen, synthesis of phytohormones, solubilization of minerals such as phosphorus, and secretion of siderophores that chelate iron and make it available to the plant root [24]. Until now there are few reports on isolation and characterization of Cd tolerant and plant growth promoting bacterial strains. However, reports related to the effect of PGPR on plant growth and physiological and biochemical aspects under the Cd stress are also rare. Keeping this in view, in the present study, bacterial strain* Bacillus cereus* has been used to investigate its growth promoting effects under the Cd^+2^stress, yet* Bacillus cereus *has been not reported as a cadmium remediater. Therefore, the objectives of the designed study were (i) to examine the role of* Bacillus cereus* as a plant growth regulator and (ii) to investigate the physiological and metabolic changes induced by* Bacillus cereus* in rice seedling under Cd stress.

## 2. Material and Methods

### 2.1. Bacterial Strain Isolation and Characterization

Cadmium resistant strain used in this study was isolated from waste water of tannery industry Peshawar, Pakistan. The bacterial isolation was carried out on the same day through the pore plate method [25] and the isolated bacteria were identified by 16S rRNA gene sequence. Briefly, DNA was isolated from the bacteria as described by [26] with minor modification. A 20 mL PCR mix was prepared containing 2*μ*L DNA, 2*μ*L 10X PCR reaction buffer, 1*μ*L dNTPs (2.5 mM) 1*μ*L forward primer (7F (5′-AGA GTT TGA TCC TGG CTCAG-3′) 1*μ*L reverse primer 492R (5′ - GGT TAC CTT GTT ACG ACT T-3′) of 10mM and 0.1 *μ*L rTaq (Takara Japan). The amplification was performed as initial denaturation for 5 mins at 95°C and followed by continuous 32 cycles of denaturation for the 30s, annealing for 30s at 56°C and extension for 72°C for 90s and finally extension at 72°C for 7 mins. The PCR product was then sequenced and was compared with the known nucleotides sequences in the Gene Bank database using BlastN (http://www.ncbi.nlm.nih.gov/BLAST). The sequencing resemblance of our isolated bacteria was 99% with* Bacillus cereus*. On the basis of resemblance with a specific group of bacteria, we conclude our bacterial isolate as a* Bacillus cereus *(Supplementary Figure 1) [27].

### 2.2. Colony Formulating Unit (CFU)

Bacterial strain was subcultured on nutrient media and was incubated for 24h at 37°C in a shaker (Wise Cube, WIS-20R) to obtain a culture density of 10^8^ CFU mL^−1^colony [13]. CFU was calculated by using the following formula:

(1) $$\begin{array}{c} CFU/mL=\frac{\left(no.\;\;of\;\;colonies\;\;\times \;\;dilution\;\;factor\right)}{volume\;\;of\;\;culture\;\;plate} \end{array}$$

### 2.3. Pot Experiment

Pot experiments were conducted to assess the effects of Cd^+2^ on the growth and physiology of rice. Seeds of* Oryza sativa *cultivars cv. Basmati 385 (B-385) and S. Basmati (S-Basmati) were collected from the National Agriculture Research Center Islamabad (NARC). The healthy and uniform seeds were surface sterilized using 3.5% sodium hypochlorite solution for 10 minutes. The sterilized seeds were placed in a 9 cm-diameter Petri dish layered with Whatman No. 1 filter paper and 10 mL distilled water was added. The Petri dishes were sealed with Parafilm and placed inside a growth chamber for germination. After germination, seven-day-old seedlings were shifted to autoclaved sand in plastic pots (1kg sand per pot) and were grown for two weeks. All pots were placed in a greenhouse with average 29 ± 1°C and 24 ± 1°C temperature for day and night, respectively, with average humidity 70% throughout the experiment. The experiment was laid out in complete randomized design (CRD) with eight treatments. According to the experimental design, 10mL bacterial suspension (10^8^ CFU) was applied to each pot, whereas a CdCl_2_ solution of different concentration, i.e., 100, 200, and 400*μ*M with and without bacterial suspension, was applied to each pot of 21-day-old seedlings [28].

### 2.4. Morphological Parameters

After 7 days of treatment, plants were harvested and were separated into shoots and roots. Root length, shoot length, and the fresh weight of plants were measured immediately after harvesting; for dry biomass, plants were placed in an oven at 80°C for 2 days.

### 2.5. Cell Membrane Stability

Cell membrane stability was calculated by using the method of Beltrano et al. [29]. Leaves were cut into small fine 1cm dices in a 20 mL test tube containing distilled water and incubated at 10°C for 24hrs. The electric-conductivity (C1) was measured and then samples were autoclaved for 15 minutes; the electroconductivity (C2) was measured after autoclaving by using conductivity meter (BMS EC Meter EC-4001). Cell membrane stability was calculated by using the formula,

(2) $$\begin{array}{c} =EC=\frac{C1}{C2}\times 100, \end{array}$$

where C refers to electroconductivity one and two.

### 2.6. Photosynthetic Pigment

Chlorophyll and carotenoid contents were measured by using the method of Lichtenthaler et al. [30]. In 5mL methanol, 25mg dried plant material along with MgO was homogenized for 2hr using an orbital shaker. The suspension was centrifuged at 3000 rpm at 25°C, and 3mL supernatant was transferred to the cuvette and absorbance was measured at three different wavelengths: 666nm, 653nm, and 470nm using spectrophotometer (UV-VIS). Various chlorophyll pigments were determined by the following formulae:

(3) Ca=15.65A666−7.340A6653Cb=27.05A653−11.21A666Cx+c=1000A470−2.860Ca−129.2Cb245

### 2.7. Micronutrients

According to Awan [31], 50mg dry material was added in 2mL sulfuric acid and 1mL of hydrogen peroxide (H_2_O_2_) and the mixture was boiled until one oily droplet was left. After cooling, 20 mL of distilled water was added and filtered by using Whatman filter paper. Ion contents (Na^+^, Ca^+2^, and K^+^) in the solution were calculated by using flame photometer (jenway pf7f).

### 2.8. Cadmium Content Determination

The methodology of Garraud et al. [32] was used for cadmium determination. Dry material 100mg was digested with sulfuric acid and nitric acid mixture and was diluted with 20mL deionized distilled water and filtered by using Whatman filter paper. Cd content in the solution was measured by using Atomic Absorption Spectroscopy (Perkin ELMER, An Analyst 4000).

### 2.9. Proline Determination

Proline content was determined according to Bates et al. [33]. Fresh plant leaves 100mg were homogenized with 5mL of 3% sulfosalicylic acid and centrifuged at 4000 rpm for 30 minutes. The supernatant of 1mL was mixed with 1mL acid ninhydrin (1.25 g ninhydrin in 30mL glacial acetic acid and 20 mL of 6 M phosphoric acid) and 1mL glacial acetic acid. The mixture was incubated for 1 hour at 100°C. The 2mL toluene was added in a mixture and was kept at room temperature until two layers became separated. The top aqueous layer was measured spectrophotometrically at an absorbance of 520nm. Proline content was determined from the standard curve and calculated by the given formula:

(4) $$\begin{array}{c} =\frac{\left(\mu g\left(proline/ml\right)\times 2/11.5\right)}{\left(0.1/5\right)} \end{array}$$

### 2.10. Total Soluble Sugar

Total soluble sugar contents were determined by the phenol-sulphuric acid method of Dey et al. [34] with slight modifications. Fresh plant material (50mg) was grounded in 3mL prewarmed 90% ethanol and incubated at 80°C for 60 min. The supernatant was transferred and the same procedure was repeated. Both supernatants were combined and added to 1:10.5% phenol, 5mL H_2_SO_4_, and 3mL distilled water under constant shaking conditions and incubated for 30 min. The absorbance was measured at 485nm using glucose as a standard.

### 2.11. Antioxidant Analysis

For antioxidant activity, 500mg fresh plant material was crushed in 10mL precooled phosphate buffer (NaH_2_PO_4_.2H_2_O 0.6663g∖L, Na_2_HPO_4_.2H_2_O 16.385g∖L) with precooled motor and pestle. After complete homogenization, the mixture was centrifuged at 20000 rpm for 20 min at 4°C as described by Vasconcelos et al. [35]. For peroxidase analysis, the 3mL reaction was prepared by adding 0.1mL 1.5% guaiacol, 0.1mL enzyme extract, and 2.7mL potassium phosphate buffer (PBS) and 0.1mL of 0.4% H_2_O_2_; after 2 to 3 mins the absorbance was noted at 470nm as mentioned by Kumar et al. [36]. Catalase activity was measured by the protocol mentioned by Vasconcelos et al. [35] in a 3 mL reaction mixture that contained 100uL 30mM H_2_O_2_, 100uL enzyme extract, and 2.8mL of 25mM potassium phosphate buffer and absorbance was determined at 240nm.

*Data Analysis*. Statistical analysis was done using SPSS version 19. Analysis of variance (ANOVA) was performed to check the significance of the different Cd doses and* B. cereus* treatment. Duncan's multiple range test was used for multiple mean comparisons. Origin 8.5 was used to plot graphs [37].

## 3. Results

### 3.1. Strain Characterization

Isolated bacterial strain was identified taxonomically by 16S rRNA gene sequence method. Results revealed that bacterial strain showed 99% similarity with Bacillus genera strain* B. cereus* 115526 by comparing with those 16S rRNA gene sequences available in the Gene Bank nucleotide sequence database (Supplementary Figure 1).

### 3.2. Seedling Vigour

**Figure 1:**
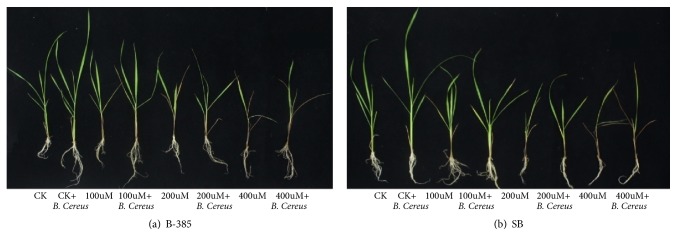
Phenotypic expression of seedling primed with* B. Cereus* on rice profile of B-385 and S. Basmati under Cd^+2^ stress. 21-day-old seedling was treated without Cd^+2^ at (100*µ*M, 200*µ*M and 400*µ*M) for 7 days and recovered for 2 days under normal condition.

Ten plants were used from three replicates to measure seedling vigour. Results showed that with the inoculation of* B. cereus *the root/shoot length and fresh and dry weights increased in both cultivars under normal as well as under stress conditions (Figure 1), while root/shoot lengths and biomass were decreased with increasing cadmium levels in both rice cultivars (B-385 and S. Basmati) but more significant decrease was noted in S. Basmati as compared to B-385.The higher root/ shoot length (17/38cm) was noted with* B. cereus *treated seedling of B-385 in control which was significantly decreased to 5/20 cm at 400*μ*M Cd^+2^level. Similarly, S. Basmati reduced 6.25/20.5cm root/shoot length under 400*μ*M stress conditions. Fresh and dry root/shoot biomasses of both cultivars were significantly increased with* B. cereus *treated seedling under control and Cd^+2^ stress conditions, whereas the maximum decrease in seedlings biomass was observed at 400uM in both rice cultivars (Table 1).

### 3.3. Electrolyte Leakage (EL)

**Figure 2:**
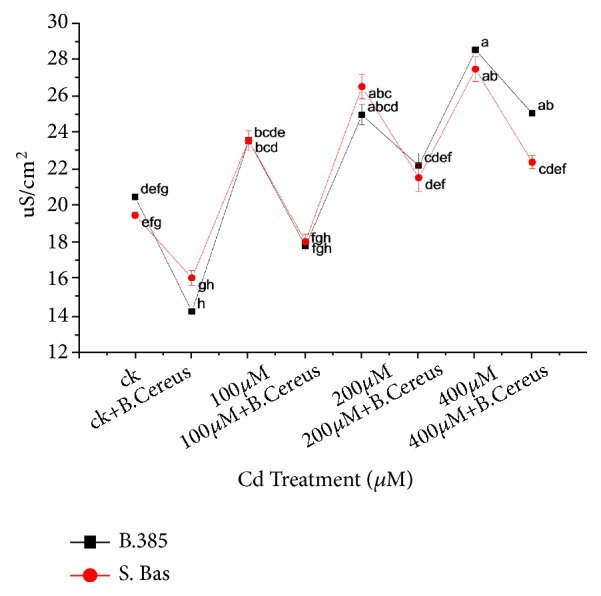
Effect of seedling primed with* B. Cereus* on Cell Membrane Stability of B.385 and S. Basmati under Cd^+2^ stress.

Electrolyte leakage was determined for the membrane permeability. Higher values of EL were recorded under 400*μ*M in both rice cultivars as compared to their controls, while the* B. cereus* treatment significantly reduced the EL values under Cd^+2^ stress in both genotypes. At 400*μ*M stress, the highest EL was observed (28.52 *μ*S/cm^2^) in B-385 and S. Basmati (27.48 *μ*S/cm^2^) which was significantly reduced to 25.96 and 22.38*μ*S/cm^2^ by* B. cereus* treatment, respectively (Figure 2).

### 3.4. Photosynthetic Pigment

Regarding the chlorophyll contents, the higher level of Cd^+2^ stress reduced chlorophyll “a” “b” and total carotenoids contents in both rice genotypes significantly. In control, the chlorophyll “a”, “b” and total carotenoids contents were 4.14, 5.38, and 395.77*μ*g/g, which were significantly reduced to 2.64, 4.69, and 279.97*μ*g/g at 400*μ*M in B-385. Seedling treatment with* B. cereus* showed promontory results under stress conditions. Similarly, S. Basmati reduced chlorophyll “a”, “b” and total carotenoids 1.85, 2.7, and 3.6 folds under 400 *μ*MCd^+2^ stress.

### 3.5. Micronutrients

Micronutrients (Na^+2^, Ca^+2^ and K^+^) were determined in both rice cultivars under control and stress conditions (Figures 4(a), 4(b), and 4(c)). Results showed that S. Basmati has significantly higher Na^+2^, Ca^+2^ ion contents as compared to B-385 under control condition. The micronutrients leakage was gradually declined with increasing Cd^+2^ content from 100*μ*M to 400*μ*M. The highest observed values of Na^+2^, Ca^+2^, and K^+^ were 15.65, 15, and 40 ppm in B-385 while 28, 18.5, and 38.5 ppm in the control of* B. cereus *treated seedlings in S. Basmati which were significantly reduced at 400*μ*M to 41, 40, and 52.5% and 44.6, 54, and 55%, respectively, while* B. cereus *treated seedlings increased the Na^+2^, Ca^+2^, and K^+^ content under stress condition in both rice cultivars.

### 3.6. Cd^+*2*^ Content

Cd^+2^ treatment plants remarkably increased the accumulation of metal in the leaves of both rice genotypes but the higher accumulation was noted in the S. Basmati seedlings (Figure 5). Results showed that Cd^+2^ contents were 10.5 and 29 folds more augmented in B-385 and S. Basmati under 400*μ*M treated seedlings as compared to control, while the seedling treated with 400*μ*M Cd^+2^ and* B. cereus *treatment reduced Cd^+2^ accumulation to 8.5 and 27.4 as compared to alone 400*μ*M Cd^+2^ stress. Seedlings treated with* B. cereus *showed alleviating results under stress conditions.

### 3.7. Proline Contents and Total Soluble Sugar (TSS)

The alone* B. cereus *application resulted in a nonsignificant reduction of proline content and significantly reduced the total soluble sugar (TSS) content in both rice cultivars under control conditions. On the other hand, the seedling treated with different concentrations of Cd^+2^ significantly increased the proline and TSS in B-385 and S. Basmati. Results showed that higher proline contents were observed in B-385 (17.4 *μ*mol/g) and S. Basmati (16.6 *μ*mol/g) at 400*μ*M which was 66 and 69% higher as compared to their respective controls. While the seedling treated with 400*μ*M and* B. cereus *reduced to 12.66 and 13.55 *μ*mol/g in B-385 and S. Basmati respectively (Figure 6). A similar trend was also noted for TSS, at 400*μ*M 2.6 and the 1.43-fold increase was noted, which was significantly reduced to 1.6 and 0.9 folds in B-385 and S. Basmati with* B. cereus *application, respectively (Figure 7).

### 3.8. Peroxidase Content

Figure 8(a) shows the effect of Cd^+2^ treatment and* B. cereus *application on POD contents under control and stress conditions in both rice cultivars. Results showed that with increasing heavy metal concentration the POD contents were also increased while the* B. cereus *applications reduced the POD content in both rice cultivars. A higher value of POD was 233.5 (U min^−1^g^−1^FW-) in B-385 while 168.12 (U min^−1^g^−1^FW-) in S. Basmati which was significantly reduced to 170.7 and 133.8 (U min^−1^g^−1^FW-) with* B. cereus *under 400 *μ*MCd^+2^ stress.

### 3.9. Catalase Activity

Catalase activity was also observed for both rice cultivars under stress conditions and results showed that CAT activity was enhanced with increasing cadmium concentration in the pots. The highest activity was observed at 400uM in both cultivars as compared to their respective control and* B. cereus *application.* B. cereus *treated seedlings showed the highest values at 400*μ*M as 1.3 -U min^−1^g^−1^FW- in B-385 and 1.2 -U min^−1^g^−1^FW- in S. Basmati which was comparatively lower than seedling untreated with* B. cereus*at400 *μ*MCd^+2^ stress (Figure 8(b)).

## 4. Discussion

Environmental pollution by heavy metals is a serious issue in most countries around the globe, which is caused by natural processes and anthropogenic activities. Among these metals, cadmium has become a serious threat due to its increasing amount in agricultural soil by several outdoor or indoor irrigation factors [38]. In agriculture soils, cadmium phytotoxicity is caused by Cd-induced oxidative stress that affects nucleic acids, proteins, and lipids, thereby causing growth inhibition or even cell death [39]. Various techniques such as adsorption, oxidation, reduction, precipitation, ion-exchange, and coagulation-flocculation have been employed for cleaning Cd-contaminated sites. However, all these practices remove the heavy metals from the soil by transforming one phase to another, which make them expensive and energy intensive [40]. Microbe assisted phytoremediation is considered as a cost-effective and environmentally friendly biotechnological approach for the remediation of heavy metals like Cd. Additionally, PGPB poses an attractive way to reduce pesticides, chemical fertilizer, and supplements inputs in agrosystem [41]. In the current study, we isolated and investigated the effect of* B. cereus *as a heavy metal remediator to alleviate the toxic effects of Cd^+2^ on rice biomass, cell membrane stability, metabolites, and antioxidants. Previously it was reported by Egidi et al. [42] that* B. cereus* can be used as a toxic metals remediator. Similarly, it was observed that inoculation of plant growth promoting bacteria (PGPB) are supposed to be highly efficient remediation method mainly for salinity [43] and heavy metals [44] without any harmful effects on soil and plant.

Cd uptake analysis (Figure 5) of the present study revealed that the accumulation of Cd increased in the roots and shoots of rice cultivars by dose-dependent manner. The application of* B. cereus* significantly decreased the uptake of metal as compared to control plants. This reduction in Cd uptake may be due to a chelating compound produced by a bacterial strain that decoys cadmium and protects the plant [45]. Khan et al. [46] also showed that wheat, maize, and all other cereals inoculated with chromium tolerant bacterial isolates lowered chromium uptake in roots and other parts of plants. The study of Jamil et al. [47] reported that* Bacillus licheniformis* (NCCP- 59) are capable to restrict Ni uptake in* Oryza sativa* L. The results of metal uptake are correlated with plant biomass reduction, as we noted that root and shoot length and biomass decreased with increasing cadmium concentration in both rice cultivars B-385 and S. Basmati (Table 1). The reduction in plant biomass might be due to cadmium that stops elongation of root/shoot fresh and dry biomasses by inhibiting cell signalling mechanism. According to Srivastava et al. [48], in soybean at 2mM cadmium stress, root and shoot length inhibited as compared to control. However, seedling treatment with* B. cereus *alleviated the drastic effects of Cd-induced toxicity on plants as shown in Table 1. Our results are in line with the findings of Luo [20], who reported that seedling inoculated with endophytic bacterium LRE07 was capable of resisting the toxic effect of heavy metal isolated from hyperaccumulator* Solanum nigrum L*. Similarly, Shirinzadeh et al. [49] also demonstrated that treatment with rhizobacteria significantly enhanced dry mass than control in barely. Similar to our findings Barassi et al. [45] reported that priming seeds with* Azospirillum brasilense* Sp245 not only enhanced seed germination but also stimulated plant growth and subset to the aerial part in lettuce when exposed to sodium chloride 80 mol m^−3^.

**Figure 3:**
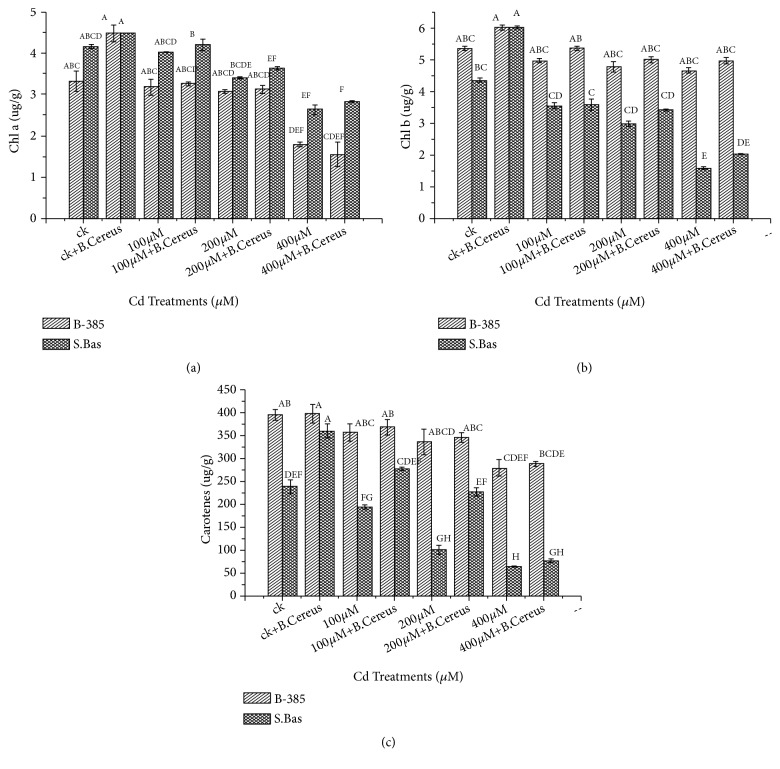
Effect of seedling primed with* B. Cereus* on Chlorophyll an (a), Chlorophyll b (b), and total carotene content (c) of B.385 and S. Basmati under Cd^+2^ stress.

Photosynthetic pigments are essential for plant survival as they act as an important component in light-harvesting complex of photo system II (PSII). Different Cadmium concentrations significantly affected chlorophyll “a” “b” and carotenoids contents by reducing pigments in green tissues (Figures 3(a)–3(c)). The decrease of photosynthetic pigment might be due to heavy metal toxicity that results in replacing magnesium ion and damaging chloroplast [45]. According to Xue et al. [50] cadmium interferes with chloroplast and photosynthetic pigments and decreases the efficiency of the carbon assimilation pathway and disturbs Chl protein complexes and Chl content [45], which results in a reduction of photosynthetic rate. Similar findings are also reported by Vijayarengan et al. [51] in rice photosynthetic pigment reduced during cadmium stress condition. However, rice plants inoculated with* B. cereus* showed maintenance of photosynthetic pigments under Cd stress (Figures 3(a)–3(c)). When rice plants are exposed to excessive levels of heavy metals, like Cd, in the growth medium, one immediate consequence involves increased generation of reactive oxygen species (ROS) within the tissues. The ROS affects cell membrane properties by causing oxidative damage to lipids, proteins, DNA, pigments, and other essential cellular molecules and can lead to a series of destructive processes [6]. Our results demonstrated that by under higher application of Cd, cell rupturing become higher in both rice cultivars (B-385 and S. Basmati) that affect osmotic regulation in the cell (Figure 2). It could be due to cadmium toxicity that disturbs the electrolytes regulation and cell membrane stringency caused leakage of electrolytes. Study of Zeid et al. [24] provide evidence that electrolyte leakage was more in alfalfa plant due to higher uptake of metals.* B. cereus *treated seedling showed effective results and lowered the electrolyte leakage with increasing cadmium content up to 400 *μ*M (Figure 2).

To cope with overproduced ROS due to the Cd uptake, plant employed ROS scavenging system consists of enzymatic and nonenzymatic antioxidants. It has been demonstrated by many researchers that antioxidants play a key role in regulating the physiological redox status and also in scavenging reactive oxygen species [48, 52]. The stimulation of antioxidants enzymes like catalase (CAT) and peroxidase (POD) acts as an essential detoxification mechanism in plants under heavy metal stresses [44]. Cadmium toxicity increases peroxidase and catalase content with increasing stress treatments in both rice cultivars (B-385 and S.B). However,* B. cereus *inoculation has significantly lowered POD and CAT activity under high cadmium concentration, which suggests that inoculation of* B. cereus *reduces oxidative stress in plants and have maintained the redox balance in stressed plants (Figure 8). Previously, Kang et al. [53] also observed that enzyme activity was lowered in paddy plants when inoculated with* Pseudomonas, pseudoalcaligenes, *and* Bacillus pumilus *in comparison to control under a saline stress condition [54].

Nonenzymatic antioxidants such as total soluble sugars, free amino acids, proline, glutathione, phenolic compounds, and ascorbic acid in plants are known to be involved in the internal detoxification of metal induce toxicity [55, 56]. Lower proline accumulation in control and higher in the stressed plant is a common phenomenon as investigated by different authors [57]. Similarly, our result suggested that proline and total soluble content increased with increasing metal toxicity, while inoculation of* B. cereus *lowered the proline and total soluble content in stressed plants (Figures 6 and 7). The lower accumulation of proline contents may be due to less accumulation of Cd, electrolyte leakage and less accumulation of ROS in the shoots. Similarly, Vardharajula [58] also showed that inoculation of* Bacillus *sp. reduces proline and total soluble content in wheat and maize as compared to untreated plants under drought stress.

**Figure 4:**
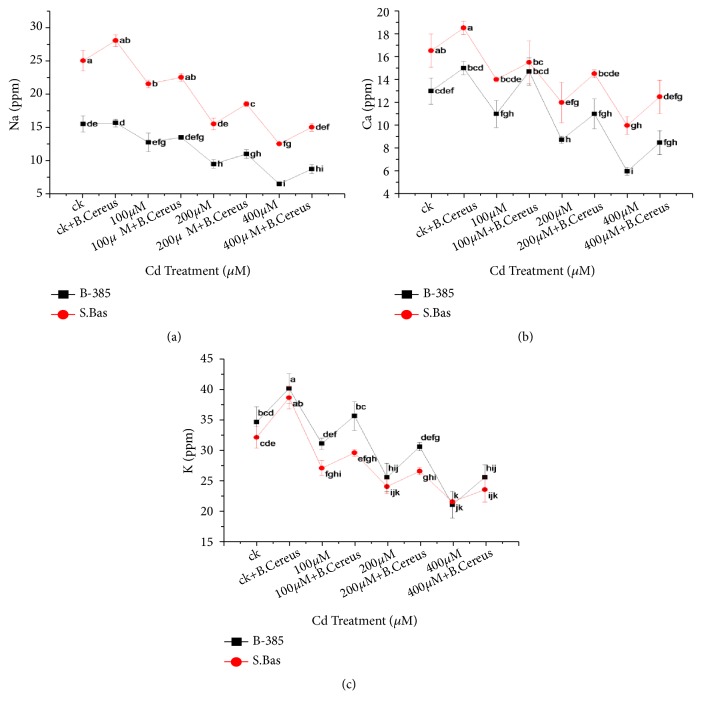
Effect of seedling primed with* B. Cereus* on Na^+2^ (a), Ca^+2^ (b), and K^+^ content (c) of B.385 and S. Basmati under Cd^+2^ stress.

**Figure 5:**
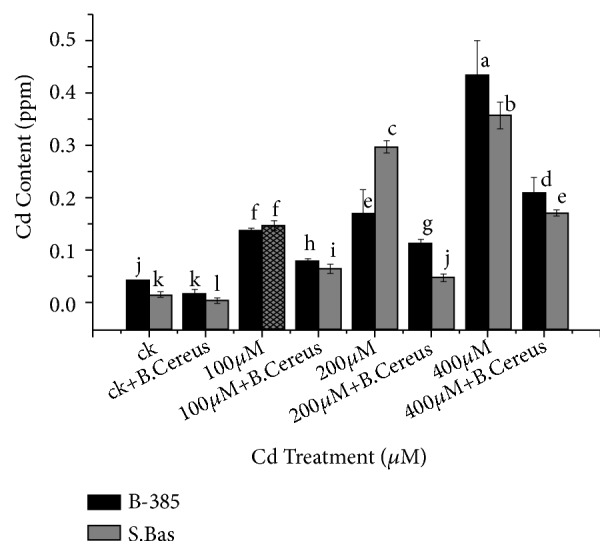
Effect of seedling primed with* B. Cereus* on Cadmium content of B.385 and S. Basmati under Cd^+2^ stress.

**Figure 6:**
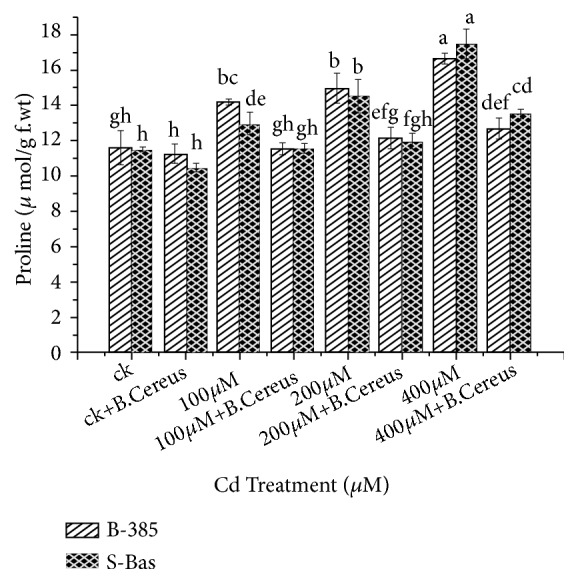
Effect of seedling primed with* B. Cereus* on proline content of B.385 and S. Basmati under Cd^+2^ stress.

**Figure 7:**
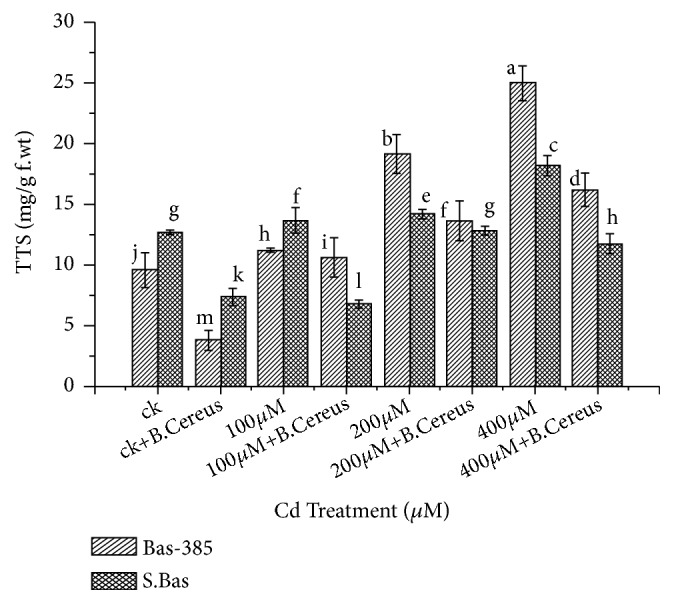
Effect of seedling primed with* B. Cereus* on Total soluble sugar(TSS) content of B.385 and S. Basmati under Cd^+2^ stress.

**Figure 8:**
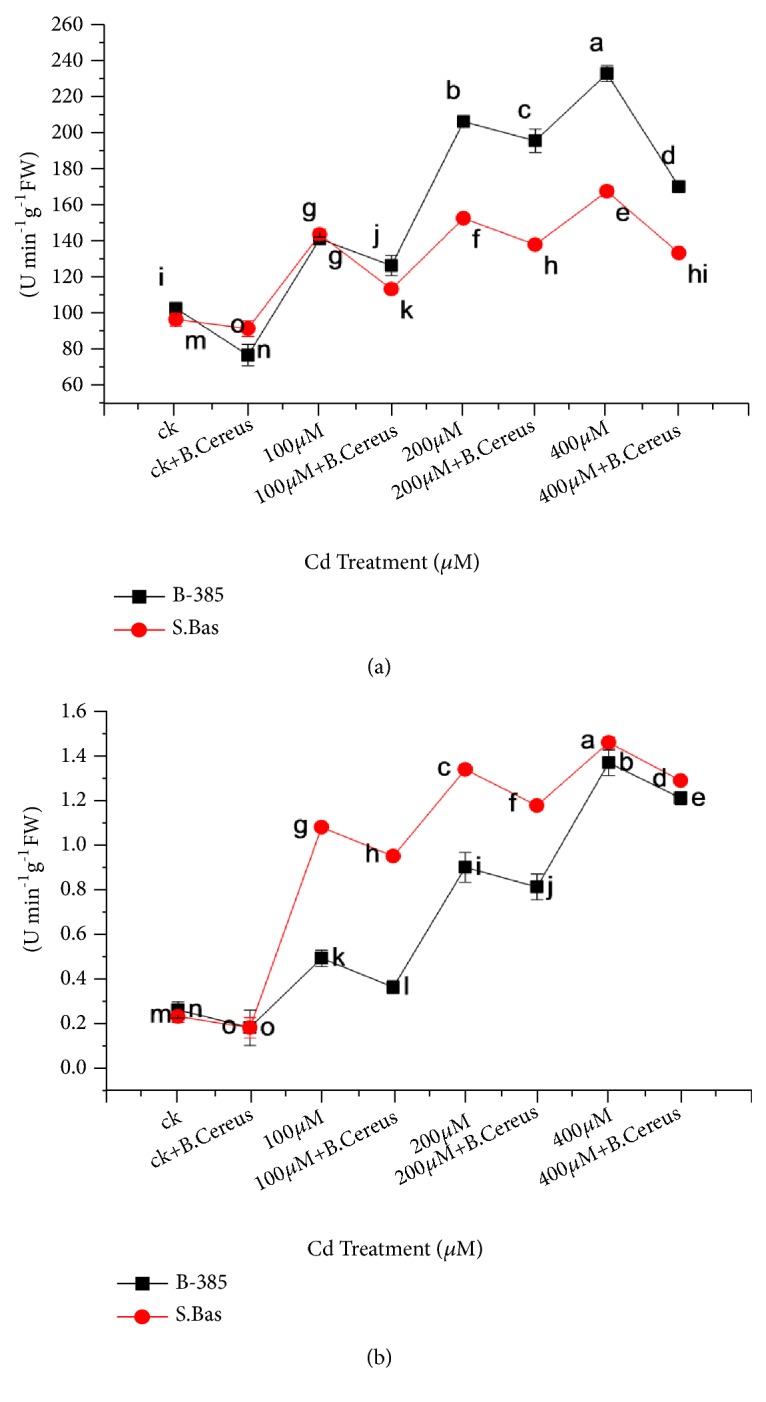
Effect of seedling primed with* B. Cereus* POD (a) and CAT content (b) of B.385 and S. Basmati under Cd^+2^ stress.

In plants, mineral nutrition, ionic balance, and acquisition are strongly affected by the cadmium presence in nutrient media. Micronutrients content considerably decreased with increasing Cd uptake in both cultivars (Figure 4). This may be due to the reason that cadmium blocked the ion channel by binding to micronutrient or causes osmotic effects due to extreme absorption in the root/leaf at a lethal level [59]. Several studies reported that cadmium decreases nutrient uptake and grain yield [49–51]. Previously, Shinwari et al. [60] demonstrated that the presence of Cr significant decrease micronutrients uptake and their distribution in rice plants. From our present results, it could be assumed that PGPB helped to maintain micronutrients in equilibrium and help the plant to overcome the competitiveness of Cd^+2^ ions [47].

## 5. Conclusion

Plant associated bacteria (PGRB) can play a beneficial role in plant growth. Nevertheless, some of these bacteria can also provide resistant to the plants against abiotic stress like heavy metal (Cd) which is not investigated in depth. Based on our knowledge,* B. cereus *can be used as plant growth promoting bacteria at the seedling stage to promote plants under high cadmium toxicity. From current studies, it can be concluded that studied bacterial stain has the capability to reduce the cadmium toxicity and enhance plant growth or it can be used in contaminated soil to overcome Cd toxicity.

## Figures and Tables

**Table 1:** Effect of seedling treated with *Bacillus cereus* physiological parameter of two rice cultivars under cadmium stress.

Cultivar	Priming	Treatments	Length		Fresh Weight		Dry Weight	
			Shoot	Root	Shoot	Root	Shoot	Root
Basmati-385	Control	Control	32.5 ± 0.883^bc^	10.5 ± 0.673^d^	0.7616 ± 0.01^de^	0.2302 ± 0.115^e^	0.192 ± 0.005^d^	0.024 ± 0.007^l^
		100 *µ*M	27.5 ± 0.98^f^	8.25 ± 0.237^ef^	0.695 ± 0.017^bc^	0.1719 ± 0.025^j^	0.161 ± 0^g^	0.021 ± 0.003^m^
		200 *µ*M	23 ± 0.784^g^	7 ± 0.023^fg^	0.6121 ± 0.005^bcd^	0.1445 ± 0.075^l^	0.142 ± 0.004^j^	0.019 ± 0.005^o^
		400 *µ*M	20.5 ± 0.93^gh^	5 ± 0.1^h^	0.5278 ± 0.01^cde^	0.1197 ± 0.105^n^	0.114 ± 0^n^	0.013 ± 0.006^p^
	*B. Cereus*	Control	38 ± 0.937^a^	17 ± 0.883^a^	1.05 ± 0.01^a^	0.304 ± 0.055^b^	0.224 ± 0.005^b^	0.032 ± 0.008^i^
		100 *µ*M	33.5 ± 0.94^bc^	12.5 ± 0.345^c^	0.84 ± 0.025^ab^	0.22 ± 0.045^f^	0.198 ± 0.009^c^	0.028 ± 0.001^j^
		200 *µ*M	28 ± 1.02^ef^	11.5 ± 0.23^ef^	0.695 ± 0.005^bc^	0.1924 ± 0.03^i^	0.179 ± 0.003^e^	0.025 ± 0.006^k^
		400 *µ*M	26.5 ± 0.938^f^	11 ± 0.17^d^	0.6232 ± 0.035^bcd^	0.1743 ± 0.04^k^	0.134 ± 0.008^k^	0.02 ± 0.004^n^

Shaheen Basmati	Control	Control	29.5 ± 0.75^de^	12.5 ± 0.373^c^	0.7011 ± 0.175^bc^	0.2366 ± 0.03^d^	0.161 ± 0.009^h^	0.034 ± 0.003^c^
		100 *µ*M	26.5 ± 0.518^f^	9.75 ± 0.198^de^	0.5263 ± 0.1^cde^	0.202 ± 0.055^h^	0.141 ± 0.004^j^	0.029 ± 0.007^e^
		200 *µ*M	25 ± 0.893^g^	8.25 ± 0.303^ef^	0.4447 ± 0.03^fg^	0.179 ± 0.045^j^	0.127 ± 0^l^	0.024 ± 0.001^f^
		400 *µ*M	20.5 ± 0.576^gh^	6.25 ± 0.029^gh^	0.3194 ± 0.03^g^	0.1371 ± 0.025^m^	0.107 ± 0.001^o^	0.02 ± 0.008^h^
	*B. Cereus*	Control	34.4 ± 0.345^b^	16 ± 0.38^b^	0.991 ± 0.025^efg^	0.295 ± 0.045^a^	0.235 ± 0.009^a^	0.038 ± 0.001^a^
		100 *µ*M	32.5 ± 1.19^bc^	14 ± 0.084^c^	0.991 ± 0.025^a^	0.279 ± 0.105^c^	0.176 ± 0.001^f^	0.031 ± 0.001^b^
		200 *µ*M	30.65 ± 0.73c^de^	9.85 ± 0.057^ef^	0.791 ± 0.03^fg^	0.212 ± 0.015^g^	0.147 ± 0.008^i^	0.027 ± 0.001^d^
		400 *µ*M	27.75 ± 0.978^ef^	8.95 ± 0.303^e^	0.55 ± 0.03^fg^	0.2 ± 0.015^h^	0.119 ± 0.009^m^	0.023 ± 0.007^g^

Each value represents the mean of three replications of each treatment. The same letters within a column indicate that there was no significant difference at a 95% probability level (p < 0.05).

## Data Availability

The data used to support the findings of this study are included within the article.

## Supplementary Materials

Supplementary Materials
* Figure S1*: Neighbor-joining phylogenetic tree of B*. cereus* as a metal (Cd) remediator based on the nucleotide sequences.
