# Supplementary material for: Bacillus Cereus Enhanced Phytoremediation Ability of Rice Seedlings under Cadmium Toxicity
Source: Biomed Res Int. 2019 Jul 24;2019:8134651. doi: 10.1155/2019/8134651 (PMC6681586; doi:10.1155/2019/8134651)
Supplement: Supplementary Materials — Figure S1: Neighbor-joining phylogenetic tree of B. cereus as a metal (Cd) remediator based on the nucleotide sequences. [file 8134651.f1.doc]

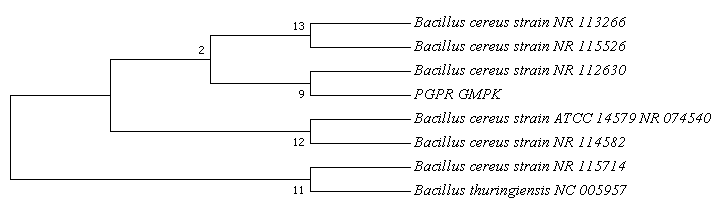


**Supplementary 1**: Evolutionary relationships of taxa.

The evolutionary history was inferred using the Neighbor-Joining method [1]. The optimal tree is shown. The tree is drawn to scale, with branch lengths in the same units as those of the evolutionary distances used to infer the phylogenetic tree. The evolutionary distances were computed using the Maximum Composite Likelihood method [2] and are in the units of the number of base substitutions per site. The analysis involved 8 nucleotide sequences. Codon positions included were 1st+2nd+3rd+Noncoding. All positions with less than 95% site coverage were eliminated. That is, fewer than 5% alignment gaps, missing data, and ambiguous bases were allowed at any position. There were a total of 692 positions in the final dataset. Evolutionary analyses were conducted in MEGA6 [3].

**References**

1. Saitou, N. and M. Nei, *The neighbor-joining method: a new method for reconstructing phylogenetic trees.* Molecular biology and evolution, 1987. 4(4): p. 406-425.

2. Tamura, K., M. Nei, and S. Kumar, *Prospects for inferring very large phylogenies by using the neighbor-joining method.* Proceedings of the National Academy of Sciences of the United States of America, 2004. 101(30): p. 11030-11035.

3. Tamura, K., et al., *MEGA6: molecular evolutionary genetics analysis version 6.0.* Molecular biology and evolution, 2013. 30(12): p. 2725-2729.
